# Gender-specific social and environmental correlates of active travel to school in four European countries: the HBSC Study

**DOI:** 10.3389/fpubh.2023.1190045

**Published:** 2023-07-25

**Authors:** Catherina Brindley, Zdenek Hamrik, Dorota Kleszczewska, Anna Dzielska, Joanna Mazur, Ellen Haug, Jaroslava Kopcakova, Adilson Marques, Teatske Altenburg, Yolanda Demetriou, Jens Bucksch

**Affiliations:** ^1^Department of Natural and Sociological Sciences, Heidelberg University of Education, Heidelberg, Germany; ^2^Department of Recreation and Leisure Studies, Faculty of Physical Culture, Palacky University Olomouc, Olomouc, Czechia; ^3^Institute of Mother and Child Foundation, Warsaw, Poland; ^4^Department of Child and Adolescent Health, Institute of Mother and Child, Warsaw, Poland; ^5^Department of Humanization in Medicine and Sexology, University of Zielona Gora, Collegium Medicum, Zielona Góra, Poland; ^6^Department of Health Promotion and Development, University of Bergen, Bergen, Norway; ^7^Department of Teacher Education, NLA University College, Bergen, Norway; ^8^Department of Health Psychology and Research Methodology, Faculty of Medicine, P.J. Safarik University, Košice, Slovakia; ^9^CIPER, Faculty of Human Kinetics, University of Lisbon, Lisbon, Portugal; ^10^Department of Public and Occupational Health, Amsterdam Public Health Research Institute, Amsterdam UMC, Vrije Universiteit Amsterdam, Amsterdam, Netherlands; ^11^Department of Sport Science, University of Innsbruck, Innsbruck, Austria

**Keywords:** active transport, students, gender, social correlates, environmental correlates, HBSC

## Abstract

**Introduction:**

Despite the health benefits, a large proportion of girls and boys in Europe do not travel to school actively. A better understanding of the correlates associated with this behavior could guide interventions. This study examines perceived social and environmental correlates of active travel to school (ACTS) from the 2017/18 Health Behavior in School-Aged Children (HBSC) survey in four European countries, with a special emphasis on gender differences (*n* = 22,023).

**Methods:**

Logistic regression was conducted to analyze associations between the perceived importance of each correlate and ACTS behavior for 11-, 13-, and 15-year-old girls and boys from Germany, Czechia, Poland, and Slovakia. All models were adjusted for age, family affluence, and meeting World Health Organization recommendations for moderate-to-vigorous physical activity.

**Results:**

Rates of ACTS significantly differed between girls and boys. In Czechia, 65% of girls and boys traveled to school actively, followed by Slovakia (61.4% girls and 58.4% boys), Poland (57.7% girls and 60.2% boys), and Germany (42.6% girls and 48.6% boys). Girls were less likely to actively travel to school compared to boys (odds ratio [OR]: 0.92, 95% confidence interval [CI]: 0.87–0.97). Increasing age (OR: 0.95, 95% CI: 0.93–0.97) and a greater distance to school index (OR: 0.89, 95% CI: 0.88–0.90) were both negatively associated with ACTS. The perceived importance of living closer to school and of road and neighborhood safety was positively associated with ACTS, with a stronger association in boys than in girls for neighborhood safety. On the contrary, the perceived importance of having people to walk with was negatively associated with ACTS, with a stronger association in girls (OR: 0.74, 95% CI: 0.65–0.84) than in boys (OR: 0.77, 95% CI: 0.66–0.88).

**Discussion:**

This study provides insights into perceived social and environmental correlates associated with ACTS behavior. Future research should include gender-specific perceptions and more in-depth investigations of correlates encouraging ACTS, especially considering social aspects, safety issues, and the structuring of the environment in different cultural settings.

## 1. Introduction

Studies show a positive association between physical activity (PA) and physical, social, psychological, and emotional health in all age groups (1, 2). Despite this, an inactive lifestyle is already observed during childhood, particularly in girls (3, 4), and continues into adolescence (5). Findings from the Health Behavior in School-Aged Children (HBSC) study show that only 23% of boys and 16% of girls meet the former World Health Organization (WHO) (6) recommendation of 60 min a day of moderate-to-vigorous physical activity (MVPA) (4, 7). Gender differences in meeting WHO PA recommendations seem to be increasing by age and to be lowest among children in northern European countries (5, 7, 8).

Active travel to school (ACTS) allows girls and boys to increase their levels of daily PA (9). Students who actively travel to school are more likely to engage in more overall PA and less sedentary behaviors and are more likely to meet PA guidelines than students who travel passively (10, 11). A study from Norway found stronger associations in girls than boys between ACTS and meeting WHO PA guidelines (12). Furthermore, several studies indicate that ACTS is positively associated with cardiovascular fitness (9, 13), self-reported weight (13), and body mass index (14) in girls and boys (9). In addition, ACTS positively affects the environment and is associated with lower greenhouse gas emissions (2).

Despite the health benefits (15), the number of walking and cycling trips undertaken by girls and boys in Europe to travel to school has declined over the last decades (16–20) or stabilized on a relatively low level (21). Compared to the United States (22) and Australia (23), rates of ACTS are still relatively high in Europe (16–19). Additionally, several studies across Europe have indicated that boys are more likely to actively travel to school compared to girls (24). However, this is not evident in all countries (21).

Social and environmental correlates of ACTS have been explored in previous studies. International studies from North America, New Zealand, and Germany show that low-income households and lower parental education are positively associated with ACTS (25–27). This social gradient has been observed in some but not all European countries (49). Furthermore, ACTS has been associated with students' individual (e.g., motivation, the company of friends or classmates) and parental (e.g., physically active and health-conscious mothers) attitudes and concerns (27, 28), as well as social and cultural norms (24). Environmental correlates of ACTS include school distance, perceived safety, walkability, traffic calming, infrastructure, recreational facilities, and urbanization (24, 29–31). As the strongest predictors of ACTS are reported the distance between home and school as well as the time required to travel to school (16, 24–27, 32). This seems even more important for secondary school students, as primary schools are often nearby, whereas students have to travel a greater distance when attending secondary school (16, 33–35).

Reasons for choosing active modes of transport show a discrepancy between and within countries, making a cross-national comparison interesting (36). Cross-national studies of modes of ACTS and associated correlates that are based on a standardized methodological approach are of interest because they can provide unique insights into how recent developments, as well as national- and local-level correlates, may have had an impact on ACTS (36). However, European findings of perceived social and environmental correlates related to ACTS from a cross-national perspective are yet lacking (37).

Studying the correlates of ACTS is an important first step in developing effective interventions to increase the number of girls and boys engaging in ACTS (38, 39). To date, it is widely recognized that gender is an important correlate of ACTS, with boys actively traveling more often to school compared to girls (13, 40, 41). However, few studies have examined gender-specific correlates of ACTS (13). One study in Germany showed that girls, but not boys, with high socioeconomic status are less likely to walk to school compared with girls with low socioeconomic status (42). Thus, to inform gender-sensitive intervention studies aimed at stimulating ACTS, more research is needed on gender differences in correlates of ACTS. However, to date, there has been a lack of studies analyzing gender differences in perceived correlates of ACTS (25, 30, 43, 44).

This study aims to identify perceived social and environmental correlates of ACTS from a European cross-national perspective, with special emphasis on differences between girls and boys.

## 2. Methods

The HBSC study is a WHO collaborative cross-sectional study conducted every 4 years, currently in 49 countries across Europe and North America. All participating countries use a standardized protocol for data collection and a standardized mandatory questionnaire (45), collecting information about health behaviors and health outcomes and social and contextual correlates of students aged 11, 13, and 15. The four countries that provided questions about ACTS and personal perceptions of environmental and social correlates of ACTS were included in the analysis. Within each country, a nationally representative random cluster sample is gathered, with units of clustering being schools and/or classes. This study represents data from the 2018 survey conducted in Czechia, Germany, Poland, and Slovakia on the optional package of ACTS.

### 2.1. Sample and data collection

A total of 22,023 (Czechia: *n* = 11,564 [49.7% girls], Germany: *n* = 4,347 [53.0% girls], Poland: *n* = 5,224 [50.8% girls], Slovakia: *n* = 888 [44.0% girls]) students were analyzed. All HBSC surveys were administered by instructed class teachers and interviewers. Participation in HBSC was voluntary, with the anonymity and confidentiality of the students ensured. Response rates for the 2017/18 HBSC study wave were 86.4% in Czechia, 53.7% in Germany, and 95% in Poland. Data from Slovakia were collected within a specific subsample of the total HBSC study sample (with special emphasis on PA), with a response rate of 81.8%. Students with complete data for all ACTS variables and covariates were included in the logistic regression.

### 2.2. Dependent variable

The use of ACTS was assessed by two questions, in which students self-reported what modes of travel they usually use to get to and from school (“walking,” “biking,” “bus, tram, subway, or boat,” “car, motorbike, and moped,” or “other means”). In line with former HBSC research (18), we decided that ACTS behavior was present if students at least walked or cycled one way to school.

### 2.3. Independent variables

#### 2.3.1. ACTS correlates

To evaluate potential correlates of ACTS, students were presented with ten different factors, frequently identified in the literature. Environmental correlates included “continuous footpaths/bicycle paths,” “wider streets or footpaths,” “less traffic,” “safe places for a bicycle at school,” “safer places to crossroad,” “access to school lockers,” “living closer to school,” and “better street lights.” Social correlates included “people to walk with” and “not worried about being bullied/attacked.” The students were asked to rate to what extent these correlates encourage them to actively travel to school. The response options were “very important,” “important,” “not important,” and “not sure.” The answer category “not important” was set as the reference category in all analyses. In the present study, the category “not sure” was omitted in the analyses as these answers cannot make a differentiated statement for perceived correlates of ACTS. The category “not sure” was selected in a minimum of 4.4% of all cases (safe places to cross the road) and a maximum of 8.5% (continuous pathways). Only 0.8% of students in the present sample selected “not sure” across all correlates.

Questions concerning social and environmental correlates of ACTS were originally derived from the School Physical Activity and Nutrition Survey (SPANS) (46).

Within HBSC, students did not report their distance to school. Therefore, to represent the distance to school, an index measuring the interaction between two items from the survey was built: the mode of transport and the duration of travel to school. Girls' and boys' transport time from home to school was assessed by the answers “ < 5 min,” “5–15 min,” “15–30 min,” “30 min to 1 h,” and “more than 1 h.” Survey items on the mode of ACTS were tested in previous research to have a correlation with the total weekday PA score, measured by accelerometers, of 0.20 (*p* < 0.01) (47). The index for distance ranges from 1 to 10 points, with “1” indicating a short duration (< 5 min) by car and “10” indicating a trip of more than 30 min by car. The index has previously been used to display an approximate measure of the location of the school in relation to the home (48).

#### 2.3.2. Covariates

The following covariates were included in all analyses: age (continuous), family affluence, distance to school index, and meeting the WHO PA recommendations.

The interaction effect between gender and the analyzed correlates was not significant. However, considering the importance of exploring gender differences in the social and environmental correlates of active travel, we decided to present the results separately for girls and boys.

Family affluence was measured using the family affluence scale (FAS). The FAS provides a measure of household material affluence among students and has previously been shown to be valid compared with a general measure of national wealth in 35 countries (r = 0.87, Cohen's kappa = 0.57) (49, 50). This scale is easy to answer for young children, applicable across countries, and based on simple indicators of affluence in the respondents' homes (50). Test–retest reliability in six countries showed a correlation of r = 0.90 (51). Four items were included in the FAS: the number of computers, car ownership, family holidays in the past year, and having one's own bedroom (49). Responses were summed into a composite score, with higher scores representing higher family affluence.

Meeting (former) WHO PA guidelines for MVPA 60 min per day was assessed by asking students, “Over the past 7 days, on how many days were you physically active for a total of at least 60 min per day?” (45). Answers ranged from 0 to 7 days. This item is well established through HBSC and showed moderate agreement (ICC = 0.6) and similarly moderate correlation (Cohen's kappa = 0.44) against a 7-day continuous measurement of total PA using an accelerometer (52). Students met the WHO recommendations when they engaged in MVPA for at least 60 min per day on 7 days within the last week (53).

### 2.4. Data analyses

Analyses were conducted with SPSS v.25 (54). The complex samples module adjusted *p*-values and 95% confidence interval (CI) for clustering effects within the primary sampling units (school class). Descriptive data for students' country, age, ACTS behavior, and time to travel to school were presented as numbers and percentages overall and for girls and boys separately. Univariate logistic regression was used to analyze the association between each correlate of ACTS and ACTS behavior, separately for girls and boys. Odds ratios (ORs) were calculated with passive commuting to school as the reference category. We tested whether correlates of ACTS varied by country by including interaction terms in each model. Multivariate logistic regressions were applied and adjusted for age, FAS, and meeting WHO MVPA recommendations.

## 3. Results

### 3.1. Socio-demographic characteristics

In total, 22,023 students from Germany (19.7%), Czechia (52.5%), Poland (23.7%), and Slovakia (4.0%) were included in the analysis (Table 1). Rates of ACTS were the highest in Czechia (65%) and the lowest in Germany (45.4%). Overall, the main part of the journey to and from school was mostly commuted by motorized means (girls 50%, boys 47.1%). Girls and boys walked to school in 43.1 and 41.2% of trips, respectively. More boys than girls biked to school (9.8 vs. 5.6%).

**Table 1:** Socio-demographic characteristics of the examined study sample^*^.

**Socio-demographic characteristics**	**Girls (50.4%)**	**Boys (49.6%)**	**Total *n* = 2,2023**
Age (years in M ± SD) *n* girls = 11,074, *n* boys = 10,909	13.4 (±1.7)	13.4 (±1.7)	13.4 (±1.7)
Number of days being physically active for at least 60 min/day in the past 7 days (M ± SD) *n* girls = 1,140, *n* boys = 10,870	**4.0 (±1.9)**	**4.4 (±2.0)**	4.2 (±2.0)
Meeting WHO recommendation of 60 min MVPA/day *n* (%) *n* girls = 11,094, *n* boys 10929, *n* total = 22,023	1,540 (13.9%)	2,287 (20.9%)	3,827 (17.4%)
**Time to travel to school** ***n*** **(%)** ***n*** **girls** = ***11,058, n boys** = **10,881***			
< 5 min	1,734 (15.7%)	2,052 (18.9%)	3,786 (17.3%)
5–15 min	5,154 (46.6%)	4,855 (44.6%)	10,009 (45.6%)
15–30 min	3,019 (27.3%)	2,740 (25.2%)	5,759 (26.3%)
30–60 min	1,006 (9.1%)	1,032 (9.5%)	2,038 (9.3%)
More than 1 h	145 (1.3%)	202 (1.9%)	347 (1.6%)
**Students actively traveling to school** ***n*** **(%)** ***n*** **girls** = ***10,964, n boys** = **10,811***	**6,427 (57.9%)**	**6,547 (59.9%)**	**12,974 (58.9%)**
Germany *n* girls = 2,186, *n* boys = 1,961 (%)	931 (42.6%)	953 (48.6%)	1,884 (45.4%)
Czechia *n* girls = 5,741, *n* boys = 5,812 (%)	3,730 (65.0%)	3,775 (65.0%)	7,505 (65.0%)
Poland *n* girls = 2,646, *n* boys = 2,545 (%)	1,526 (57.7%)	1,531 (60.2%)	3,057 (58.9%)
Slovakia *n* girls = 391, *n* boys = 492 (%)	240 (61.4%)	288 (58.4%)	528 (59.7%)

n, sample size; M, mean; SD, standard derivation; WHO, World Health Organization; MVPA, moderate-to-vigorous physical activity. ^*^Significant gender differences on α = 95% are bolded; *n* differs for the variables due to missing values.

Girls were less likely to actively travel to school compared to boys (OR 0.92, 95% CI 0.87–0.97). Increasing age (OR 0.95, 95% CI 0.93–0.97) and a greater distance to school index (OR 0.89, 95% CI 0.88–0.90) were both negatively associated with ACTS. A positive association was found between meeting the WHO PA recommendation (OR 1.20, 95% CI 1.11-1.29) as well as the number of days per week being 60 min physically active (OR 1.05, 95% CI 1.03–1.06) and ACTS. A high family affluence compared to a low family affluence was negatively associated with ACTS across all countries (OR 0.47, 95% CI 0.44–0.51) (Supplementary material 1).

Social and environmental correlates showed significant associations with ACTS across all countries when analyzed univariate (Supplementary material 1), except for “wide pavements/footpaths” (very important ratings), “safe places to cross road” (important ratings), and “better street lights” (all ratings). Interaction terms between correlates of ACTS and country were significant except for “living closer to school” and “better street lights,” indicating that correlates of ACTS differ by country for both girls and boys (results not shown). Therefore, the overall results and the results for each country are stratified by boys and girls.

### 3.2. ACTS correlates in girls

**Figure 1:**
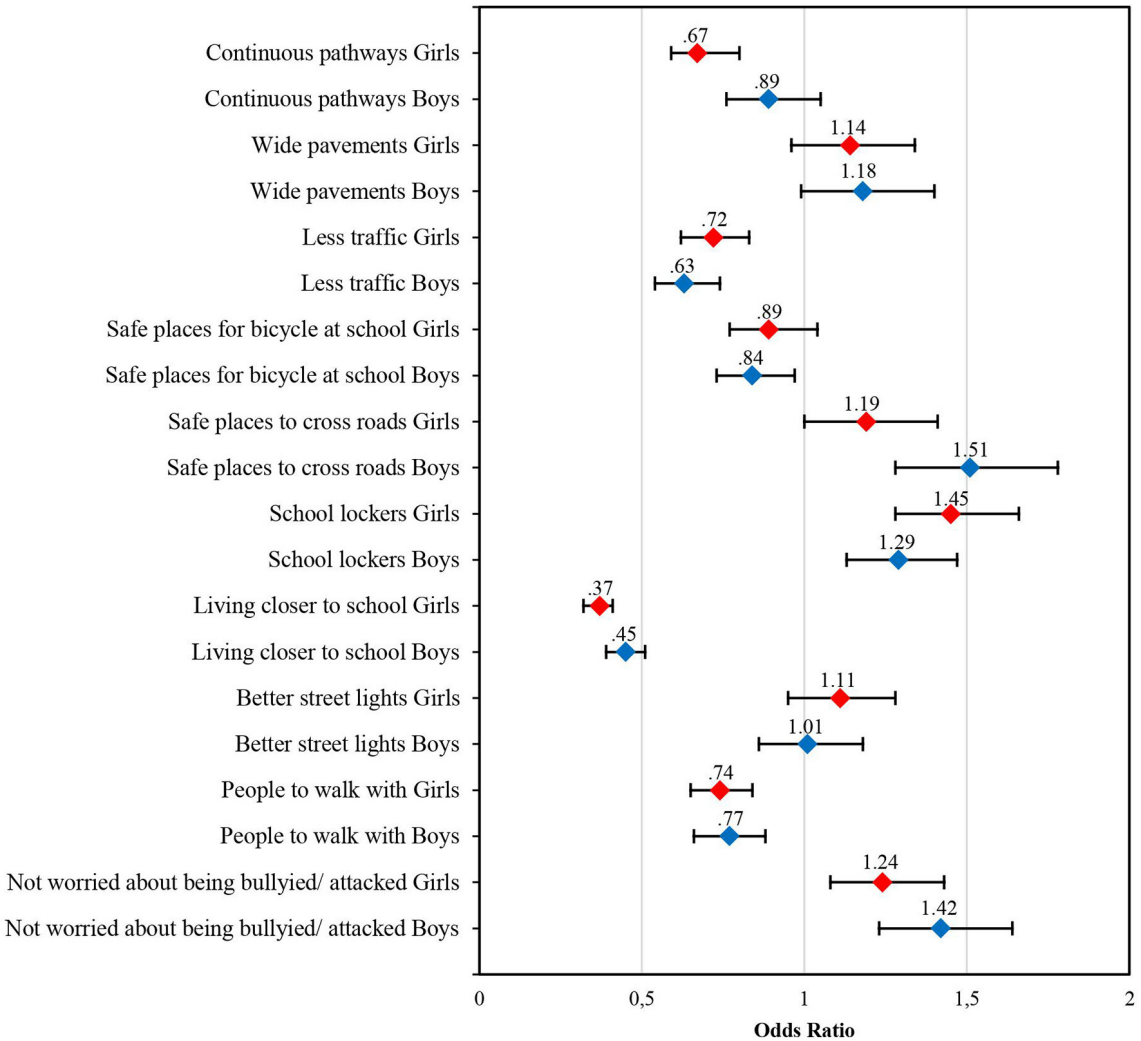
Multivariate odds ratio and 95% confidence intervals of girls and boys very important perceptions of 10 environmental and social ACTS correlates (all countries).

Girls who reported “living closer to school” as an important or very important encouraging factor for walking or cycling were less likely to ACTS (Table 2). Similarly, those who reported “continuous pathways,” “less traffic,” and “safe places for bicycle at school” as very important or important correlates for making walking or cycling to school better were less likely to ACTS. A positive association with ACTS was found in girls who rated having a “school locker” as very important. Regarding social correlates of ACTS, girls who rated it as very important or important to have “people to walk with” to school were less likely to travel to school by foot or by bike. In contrast, girls were more likely to ACTS when they rated “not worried about being bullied or attacked” as important (Figure 1).

**Table 2:** Multivariate logistic regression of 10 perceived correlates in relation to ACTS for girls (0 = passive, 1 = active)^*^.

	**Overall** ***n** =* **8,113**			**Germany** ***n** =* **2,004**			**Czechia** ***n** =* **3,330**			**Poland** ***n** =* **2,500**			**Slovakia** ***n** =* **279**		
**Variables**	**OR**	**95% CI**	**p**	**OR**	**95% CI**	**p**	**OR**	**95% CI**	**p**	**OR**	**95% CI**	**p**	**OR**	**95% CI**	**p**
**(1) CONTINUOUS PATHWAYS**															
(a) Not important (ref.)	1	-	-	1	-	-	1	-	-	1	-	-	1	-	-
(b) Important	**0.75**	**0.66–0.85**	**<0.001**	0.89	0.69–1.15	0.36	**0.67**	**0.55–0.82**	**<0.001**	1.02	0.77–1.37	0.88	0.48	0.22–1.05	0.07
(c) Very important	**0.67**	**0.59–0.80**	**<0.001**	1.28	0.94–1.75	0.12	**0.42**	**0.32–0.55**	**<0.001**	0.92	0.67–1.27	0.63	0.91	0.30–2.74	0.87
**(2) Wide pavements/footpaths**															
(a) Not important (ref.)	1	-	-	1	-	-	1	-	-	1	-	-	1	-	-
(b) Important	0.98	0.87–1.10	0.76	0.87	0.70–1.08	0.21	1.06	0.88–1.28	0.53	0.93	0.72–1.21	0.61	0.58	0.29–1.18	0.13
(c) Very important	1.14	0.96–1.34	0.13	1.15	0.83–1.58	0.41	1.20	0.90–1.61	0.21	0.95	0.69–1.31	0.77	0.38	0.11–1.24	0.11
**(3) Less traffic**															
(a) Not important (ref.)	1	-	-	1	-	-	1	-	-	1	-	-	1	-	-
(b) Important	**0.85**	**0.76–0.96**	**0.01**	0.86	0.68–1.09	0.21	0.86	0.71–1.04	0.12	0.85	0.67–1.07	0.16	1.05	0.51–2.15	0.90
(c) Very important	**0.72**	**0.62–0.83**	**<0.001**	0.76	0.57–1.02	0.07	**0.71**	**0.56–0.90**	**0.005**	**0.69**	**0.52–0.91**	**0.01**	0.98	0.38–2.57	0.97
**(4) Safe places for bicycles at school**															
(a) Not important (ref.)	1	-	-	1	-	-	1	-	-	1	-	-	1	-	-
(b) Important	**0.81**	**0.70–0.93**	**0.003**	0.97	0.71–1.31	0.82	0.83	0.67–1.02	0.08	0.76	0.58–1.01	0.06	**0.34**	**0.14–0.87**	**0.02**
(c) Very important	0.89	0.77–1.04	0.14	1.10	0.80–1.52	0.56	0.93	0.74–1.18	0.57	0.88	0.65–1.18	0.38	0.60	0.22–1.65	0.32
**(5) Safe places to cross the road**															
(a) Not important (ref.)	1	-	-	1	-	-	1	-	-	1	-	-	1	-	-
(b) Important	1.17	1.00–1.37	0.05	1.11	0.81–1.53	0.52	1.23	0.97–1.56	0.09	1.21	0.89–1.65	0.22	0.60	0.23–1.59	0.30
(c) Very important	1.19	1.00–1.41	0.05	1.14	0.80–1.63	0.47	1.17	0.90–1.53	0.26	1.22	0.87–1.71	0.25	1.55	0.49–4.89	0.45
**(6) School lockers**															
(a) Not important (ref.)	1	-	-	1	-	-	1	-	-	1	-	-	1	-	-
(b) Important	**1.15**	**1.01–1.31**	**0.03**	**0.79**	**0.63–0.99**	**0.04**	1.15	0.91–1.44	0.25	1.27	0.95–1.69	0.11	0.79	0.33–1.90	0.60
(c) Very important	**1.45**	**1.28–1.66**	**<0.001**	**0.67**	**0.51–0.88**	**0.003**	**1.52**	**1.20–1.93**	**<0.001**	**1.49**	**1.12–1.99**	**0.01**	0.94	0.35–2.51	0.90
**(7) Living closer to school**															
(a) Not important (ref.)	1	-	-	1	-	-	1	-	-	1	-	-	1	-	-
(b) Important	**0.72**	**0.64–0.80**	**<0.001**	0.81	0.65–1.03	0.08	**0.64**	**0.53–0.78**	**<0.001**	**0.73**	**0.59–0.91**	**0.004**	0.70	0.35–1.40	0.31
(c) Very important	**0.37**	**0.32–0.41**	**<0.001**	**0.41**	**0.31–0.54**	**<0.001**	**0.31**	**0.25–0.38**	**<0.001**	**0.37**	**0.30–0.46**	**<0.001**	0.55	0.22–1.37	0.20
**(8) Better street lights**															
(a) Not important (ref.)	1	-	-	1	-	-	1	-	-	1	-	-	1	-	-
(b) Important	1.02	0.90–1.15	0.79	1.06	0.33–1.85	0.58	1.02	0.84–1.24	0.82	0.77	0.60–1.00	0.05	1.95	0.92–4.17	0.08
(c) Very important	1.11	0.95–1.28	0.18	0.90	0.67–1.20	0.47	1.23	0.96–1.59	0.10	0.79	0.60–1.05	0.11	2.09	0.77–5.68	0.15
**(9) People to walk with**															
(a) Not important (ref.)	1	-	-	1	-	-	1	-	-	1	-	-	1	-	-
(b) Important	**0.74**	**0.66–0.83**	**<0.001**	**0.64**	**0.50–0.81**	**<0.001**	**0.80**	**0.66–0.96**	**0.02**	0.98	0.76–1.26	0.87	1.75	0.84–3.66	0.13
(c) Very important	**0.74**	**0.65–0.84**	**<0.001**	**0.51**	**0.39–0.65**	**<0.001**	**0.76**	**0.60–0.96**	**0.02**	1.21	0.93–1.56	0.16	0.86	0.39–1.91	0.71
**(10) Not worried about being bullied/attacked**															
(a) Not important (ref.)	1	-	-	1	-	-	1	-	-	1	-	-	1	-	-
(b) Important	**1.15**	**1.01–1.32**	**0.04**	1.16	0.87–1.54	0.31	1.14	0.92–1.41	0.22	1.14	0.88–1.48	0.32	1.98	0.89–4.38	0.09
(c) Very important	**1.24**	**1.08–1.43**	**0.003**	1.27	0.97–1.68	0.09	**1.33**	**1.05–1.68**	**0.02**	1.26	0.96–1.65	0.10	**2.56**	**1.06–6.20**	**0.04**

*n*, sample size; OR, odds ratio; CI, confidence interval; ref., reference category. ^*^Adjusted for age, FAS, meeting WHO PA recommendations, and distance to school. Significant results on α = 95 % are **bolded**.

### 3.3. Country-specific ACTS correlates in girls

None of the examined correlates was correlated with ACTS for all countries. For girls from Germany, Poland, and Czechia, rating “living closer to school” as very important to improve walking or cycling was negatively associated with ACTS, while rating “school lockers” as very important was positively associated with ACTS. For Slovakian girls, very important perceptions of “not being worried about being bullied or attacked” and important perceptions of “safe places for bicycle at school” were positive correlates of ACTS (Table 2).

### 3.4. ACTS correlates in boys

Boys were less likely to ACTS when they indicated that “living closer to school” was important or very important (Table 3). Additionally, boys who perceived “less traffic” as important or very important, “continuous pathways” as important, or “safe places for bicycle at school” as very important to encourage ACTS were less likely to travel to school by foot or bike. Boys who perceived “safe place to cross roads” and having a “school locker” as very important to make walking or biking better showed a positive association with ACTS. Concerning social correlates, very important perceptions of “people to walk with” were associated with less ACTS, whereas the same ratings of “not worried about being bullied or attacked” were positively associated with ACTS (Figure 1).

**Table 3:** Multivariate logistic regression of 10 perceived correlates in relation to ACTS for boys (0 = passive, 1 = active)^*^.

	**Overall** ***n** =* **7,826**			**Germany** ***n** =* **1,763**			**Czechia** ***n** =* **3,372**			**Poland** ***n** =* **2,355**			**Slovakia** ***n** =* **336**		
**Variables**	**OR**	**95% CI**	**p**	**OR**	**95% CI**	**p**	**OR**	**95% CI**	**p**	**OR**	**95% CI**	**p**	**OR**	**95% CI**	**p**
**(1) Continuous pathways**															
(a) Not important (ref.)	1	-	-	1	-	-	1	-	-	1	-	-	1	-	-
(b) Important	**0.82**	**0.72–93**	**0.002**	1.21	0.93–1.58	0.16	**0.71**	**0.58–0.87**	**0.001**	0.79	0.60–1.04	0.09	1.36	0.70–2.65	0.37
(c) Very important	0.89	0.76–1.05	0.16	**1.52**	**1.12–2.01**	**0.01**	**0.70**	**0.54–0.91**	**0.009**	0.90	0.66–1.24	0.52	0.85	0.38–1.91	0.70
**(2) Wide pavements/footpaths**															
(a) Not important (ref.)	1	-	-	1	-	-	1	-	-	1	-	-	1	-	-
(b) Important	0.89	0.78–1.01	0.06	0.91	0.71–1.17	0.47	0.91	0.75–1.11	0.36	0.81	0.62–1.05	0.11	1.13	0.60–2.11	0.70
(c) Very important	1.18	0.99–1.40	0.07	1.16	0.83–1.62	0.38	**1.63**	**1.18–2.24**	**0.003**	0.95	0.68–1.32	0.76	0.64	0.27–1.56	0.33
**(3) Less traffic**															
(a) Not important (ref.)	1	-	-	1	-	-	1	-	-	1	-	-	1	-	-
(b) Important	**0.76**	**0.68–0.86**	**<.001**	**0.74**	**0.58–0.95**	**0.02**	**0.67**	**0.55–0.81**	**<.001**	0.93	0.74–1.18	0.57	0.61	0.32–1.15	0.13
(c) Very important	**0.63**	**0.54–0.74**	**<.001**	**0.66**	**0.48–0.90**	**0.01**	**0.53**	**0.41–0.68**	**<.001**	**0.70**	**0.53–0.93**	**0.01**	0.87	0.37–2.04	0.75
**(4) Safe places for bicycles at school**															
(a) Not important (ref.)	1	-	-	1	-	-	1	-	-	1	-	-	1	-	-
(b) Important	0.88	0.76–1.02	0.09	1.02	0.75–1.39	0.90	0.85	0.68–1.07	0.17	1.11	0.84–1.46	0.46	0.61	0.29–1.27	0.19
(c) Very important	**0.84**	**0.73–0.97**	**0.02**	1.34	0.97–1.85	0.07	**0.65**	**0.52–0.81**	**<.001**	1.10	0.83–1.45	0.51	0.66	0.31–1.41	0.29
**(5) Safe places to cross the road**															
(a) Not important (ref.)	1	-	-	1	-	-	1	-	-	1	-	-	1	-	-
(b) Important	**1.23**	**1.07–1.42**	**0.003**	1.18	0.88–1.59	0.27	**1.28**	**1.03–1.58**	**0.02**	1.22	0.93–1.60	0.14	0.72	0.32–1.62	0.42
(c) Very important	**1.51**	**1.28–1.78**	**<.001**	1.27	0.90–1.80	0.17	**1.65**	**1.27–2.14**	**<.001**	**1.47**	**1.08–2.00**	**0.02**	1.18	0.47–3.00	0.72
**(6) School lockers**															
(a) Not important (ref.)	1	-	-	1	-	-	1	-	-	1	-	-	1	-	-
(b) Important	1.13	1.00–1.28	0.06	**0.77**	**0.60–0.99**	**0.04**	1.08	0.87–1.34	0.49	**1.31**	**1.02–1.69**	**0.04**	1.17	0.60–2.28	0.64
(c) Very important	**1.29**	**1.13–1.47**	**<.001**	1.06	0.78–1.45	0.72	1.19	0.95–1.50	0.13	**1.42**	**1.10–1.84**	**0.01**	0.85	0.40–1.79	0.67
**(7) Living closer to school**															
(a) Not important (ref.)	1	-	-	1	-	-	1	-	-	1	-	-	1	-	-
(b) Important	**0.79**	**0.70–0.90**	**<.001**	1.00	0.78–1.29	0.99	**0.71**	**0.58–0.86**	**0.001**	**0.74**	**0.59–0.92**	**0.01**	0.87	0.46–1.64	0.67
(c) Very important	**0.45**	**0.39–0.51**	**<.001**	**0.40**	**0.30–0.54**	**<.001**	**0.40**	**0.32–0.50**	**<.001**	**0.45**	**0.35–0.57**	**<.001**	1.22	0.59–2.54	0.60
**(8) Better street lights**															
(a) Not important (ref.)	1	-	-	1	-	-	1	-	-	1	-	-	1	-	-
(b) Important	0.95	0.83–1.07	0.37	0.99	0.77–1.27	0.94	1.08	0.88–1.31	0.46	0.79	0.62–1.02	0.07	0.72	0.39–1.36	0.31
(c) Very important	1.01	0.86–1.18	0.93	1.06	0.76–1.42	0.73	0.96	0.72–1.26	0.76	0.88	0.66–1.16	0.37	0.99	0.42–2.30	0.97
**(9) People to walk with**															
(a) Not important (ref.)	1	-	-	1	-	-	1	-	-	1	-	-	1	-	-
(b) Important	**0.81**	**0.72–0.91**	**<.001**	**0.73**	**0.57–0.93**	**0.01**	1.04	0.84–1.28	0.75	**0.73**	**0.58–0.92**	**0.01**	0.97	0.53–1.77	0.92
(c) Very important	**0.77**	**0.66–0.88**	**<.001**	**0.60**	**0.46–0.80**	**<.001**	1.07	0.79–1.46	0.65	0.82	0.64–1.06	0.13	**0.43**	**0.20–0.96**	**0.04**
**(10) Not worried about being bullied/attacked**															
(a) Not important (ref.)	1	-	-	1	-	-	1	-	-	1	-	-	1	-	-
(b) Important	**1.19**	**1.04–1.35**	**0.01**	1.15	0.86–1.53	0.35	**1.26**	**1.01–1.55**	**0.04**	1.02	0.80–1.30	0.87	1.93	0.98–3.81	0.06
(c) Very important	**1.42**	**1.23–1.64**	**<.001**	**1.43**	**1.08–1.88**	**0.01**	**1.35**	**1.06–1.71**	**0.02**	**1.39**	**1.06–1.83**	**0.02**	**2.38**	**1.06–5.36**	**0.04**

*n*, sample size; OR, odds ratio; CI, confidence interval; ref., reference category. ^*^Adjusted for age, FAS, meeting WHO PA recommendations, and distance to school. Significant results on α = 95% are **bolded**.

### 3.5. Country-specific ACTS correlates in boys

The environmental correlates “living closer to school” and “less traffic” were associated with ACTS for German, Czech, and Polish boys but not for Slovakian boys (Table 3). Only for Czech boys, very important perceptions of having “wide pavements” and “safe places for bicycle at school” to encourage walking or cycling were positively associated with ACTS. German boys who found it very important to have “continuous pathways” on their way to school were more likely to actively travel to school, while indications of “less traffic,” “school lockers,” and “people to walk with” revealed a negative association with ACTS.

The social correlate of “not being worried about being bullied or attacked” on the way to school showed a positive association with ACTS across all countries (Table 3). Boys who perceived this item as very important were more likely to actively travel to school. The strongest association for this item was found for Slovakian boys, while correlations were similar for Germany, Czechia, and Poland. Only for Slovakian boys, “people to walk with” was negatively associated with ACTS (Table 3).

## 4. Discussion

In our study, rates of ACTS were highest in Czechia (65%) followed by Slovakia (61%), Poland (58%), and Germany (43%), and overall, girls had lower rates than boys (58 and 60%, respectively). We found that students who indicated that living closer to school would be very important for encouraging walking or cycling to school were less likely to actively travel to school across all countries and for girls and boys. Students who perceived road and neighborhood safety as important to encourage walking or cycling were less likely to travel to school actively. This perception was stronger in boys compared to girls. Girls and boys who perceived having people to walk with as important had less ACTS. This association was stronger in girls in comparison to boys. “Not worried about being bullied or attacked” was positively associated with ACTS, with a higher correlation in boys than in girls.

A recent study demonstrated that rates of ACTS decreased from about 70% in 2006 to approximately 50% in 2018 in Czechia (20). In our study, 65% of students from Czechia traveled to school actively, which represents a higher rate of ACTS. For Germany and Poland, rates of ACTS are in line with former research, where approximately 50% of students actively travel to school (27, 55–57). In the present study, ACTS rates significantly differed between girls and boys in all countries, which was in line with data from the HBSC questionnaire 2003/2004 in Slovakia (58), but in contrast to other studies (21, 27, 59, 60).

Previous studies have demonstrated that distance is one of the strongest correlates of ACTS (16, 30). In our study, students were less likely to actively travel to school when a short distance between school and home was important to them. This was in contrast to the actual correlation between distance and ACTS in our data: students living closer to school had higher ACTS levels. This finding is very interesting and highlights the importance of objective measurement of distance as well as the subjective perceptions of students.

Previous studies indicated that the built environment on the way to school is another important aspect that influences whether students choose an active way of commuting (61). In our study, the perceived importance of “less traffic” and “safe places to cross roads” to make walking or cycling better confirmed this, as they were positively associated with ACTS. Additionally, both correlates were more strongly associated with ACTS in boys than in girls. In the current study, a higher proportion of boys than girls reported using a cycle as a mode of transportation to school. As cycling is a faster but more dangerous way of commuting, these traffic aspects might be more important for cycling. In line with this hypothesis, a previous study on correlates of cycling in students indicated that in boys (but not girls), school neighborhood design was significantly associated with cycling, that is, boys attending schools in neighborhoods with high connectivity and low traffic were 5.6 times more likely to cycle (95% CI 1.11–27.96) and for each kilometer boys lived from school, the odds of cycling decreased by 0.70 (95% CI 0.63–0.99) (62). Furthermore, we found that perceptions of “continuous pathways” were more strongly associated with ACTS in girls than in boys. As continuous pathways are more important for walking than cycling, this finding might imply that in our study, girls have higher walking rates to school than boys. However, this hypothesis needs to be confirmed in future research. We would therefore recommend studying perceptions of ACTS correlates specific to the mode of transportation.

Our study demonstrated that girls and boys who perceived road and neighborhood safety as important for ACTS were less likely to actively travel to school, with slightly higher associations in girls compared to boys. Recent studies demonstrated that students, especially those with longer commuting distances, depend on their choice of ACTS on how safe they perceive the environmental aspects of the way to school (63). The results from research, mostly based on parental perceptions, concluded that safety issues are important correlates influencing ACTS, especially in girls (64, 65). Our findings reflecting students' perceptions thus align with the findings of parental perceptions on ACTS (28).

Our analysis revealed that girls and boys who perceived “not worried about being bullied or attacked” as important had higher odds of active commuting across all countries. Our findings suggest that students' fear of being bullied or attacked on their way to school may negatively impact their ACTS. We recommend future studies to further explore safety perceptions in girls and boys and their association with ACTS.

In our study, we found that none of the examined correlates in girls were consistently associated with ACTS in all countries. Overall, road and neighborhood safety items were correlates of students' ACTS in our study. However, in our sample, these correlates were not significant for all countries. Moreover, in the case of significant correlates, the direction and strength of the associations varied by country. The cross-country results indicate that correlates of ACTS differ by country and highlight the importance of analyzing correlates of ACTS in a country-specific manner.

In our sample, perceiving having “people to walk with” as important for encouraging ACTS reduced the odds of ACTS. Social norms, social modeling, and social support are defined as the most consistent correlates influencing ACTS (66). Also fulfilling the need for relatedness, according to the literature, is a motivating factor for ACTS (67). It is to be determined if our result derives from existing friendship agreements for walking and cycling to school together. This might have led to the result that having people to walk or cycle to school with is a matter of course for the students in our sample and therefore not perceived as important to encourage ACTS.

### 4.1. Strengths and limitations

The strengths of the current study are its large sample size, the representative international dataset, and the cross-country-and gender-specific comparison of social and environmental correlates of ACTS. To the best of our knowledge, cross-national studies in Europe were lacking. Little was known about the association between correlations of ACTS and gender.

However, some limitations have to be taken into account when interpreting the results. Due to the cross-sectional design of the HBSC study, conclusions about causality cannot be drawn. Additionally, we found hardly any significant associations with ACTS for students from Slovakia, which might be due to a low number of participants. Furthermore, the lack of standardized measures of correlates of ACTS and comparable control variables across different studies makes it difficult to compare and interpret findings (14, 25, 68, 69). In line with this, it is important to take into account how correlates in this study have been assessed. Although self-reported assessments of correlates do not overall agree with objective measurements (70), previous research has demonstrated that people's subjective perception of their environment is strongly correlated to their probability to act (61). This study makes an important contribution to the field by addressing the research gap in the association of perceived correlates with ACTS for different regional locations (55, 71). Furthermore, the distance from home to school was not collected objectively. However, the index for distance from home to school was included in the analysis and showed a positive association with ACTS.

For this study, the former version of WHO PA recommendations was used, as the survey collection was before the new guidelines in 2020 were released. The new guidelines could allow more girls and boys to meet WHO PA recommendations as weekly PA time is summed up instead of being active for at least 60 min per day. Future studies are needed to analyze if the association between ACTS and PA is still consistent with the recently released recommendations (53).

### 4.2. Conclusion

This study provides insight into perceived environmental and social correlates that might encourage students' ACTS in and across four European countries, with special emphasis on gender differences. The perceived importance of living closer to school was a strong correlate of ACTS in boys and girls, with higher importance ratings associated with less ACTS. In girls, having school lockers and continuous pathways were the two most important correlates associated with ACTS. In boys, road and neighborhood safety correlates showed a positive association with ACTS and were most important. The importance of having people to walk with showed a higher negative association with ACTS in girls compared to boys. Cross-country-specific results revealed that for girls, having a school locker and living closer to school are associated with ACTS in all countries except Slovakia. For boys, less traffic and living closer to school are associated with ACTS in all countries except Slovakia. In all countries, not being worried about being bullied or attacked is perceived as important and is correlated with ACTS.

Future studies should take gender-specific perceptions of correlates encouraging ACTS into account when developing and implementing intervention programs and public health policies aimed at increasing the percentage of school-aged children who actively travel to and from school. Decision makers for public health and school policies, as well as intervention developers, should consider the importance of safe infrastructure and continuous pathways (72), lockers in schools, and the benefits of encouraging young people to travel to school together as key elements (6, 73). Furthermore, intervention programs prepared as a part of intervention and/or policy development should also address social and neighborhood features. Therefore, further research could also investigate perceptions of the potential influence of other social and environmental correlates on ACTS behavior, such as public transportation accessibility and parental attitudes toward active transportation.

## Data availability statement

The datasets presented in this study can be found in online repositories. The names of the repository/repositories and accession number(s) can be found below: https://www.uib.no/en/hbscdata/113290/open-access.

## Ethics statement

Ethical review and approval was not required for the study on human participants in accordance with the local legislation and institutional requirements. Written informed consent to participate in this study was provided by the participants' legal guardian/next of kin.

## Author contributions

CB: data analysis and original draft writing and preparation. JB: supervision. YD: project administration and funding acquisition. ZH, DK, AD, JM, EH, JK, and JB: participation in data collection. All authors have contributed to the manuscript and approved the submitted version.
