# Supplementary material for: Gender-specific social and environmental correlates of active travel to school in four European countries: the HBSC Study
Source: Front Public Health. 2023 Jul 25;11:1190045. doi: 10.3389/fpubh.2023.1190045 (PMC10407096; doi:10.3389/fpubh.2023.1190045)
Supplement: Supplementary file 1 [file Table_1.DOCX]

Supplementary Material

Gender-specific social and environmental correlates of active travel to school in four European countries: the HBSC Study

Catherina Brindley*, Zdenek Hamrik, Dorota Kleszczewska, Anna Dzielska, Joanna Mazur, Ellen Haug, Jaroslava Kopcakova, Adilson Marques, Teatske Altenburg, Yolanda Demetriou, Jens Bucksch

*** Correspondence:** Catherina Brindley: [brindley@ph-heidelberg.de](mailto:brindley@ph-heidelberg.de)

**Supplementary Table 1:** Associations (univariate) of social and environmental correlates with students’ ACTS

| **Variable** | **OR** | **95% CI (α=95%)** | **p (α=95%)** |
| --- | --- | --- | --- |
| 1) CONTINUOUS PATHWAYS |  |  |  |
| a) not important (ref.) | 1 | - | - |
| b) important | **.76*** | **.70 - .82** | **.00** |
| c) very important | **.78*** | **.72 - .85** | **.00** |
| 2) WIDE PAVEMENTS / FOOTPATHS |  |  |  |
| a) not important (ref.) | 1 | - | - |
| b) important | **.89*** | **.83 - .96** | **.00** |
| c) very important | .97 | .89 -1.07 | .57 |
| 3) LESS TRAFFIC |  |  |  |
| a) not important (ref.) | 1 | - | - |
| b) important | **.81*** | **.76 - .87** | **.00** |
| c) very important | **.69*** | **.64 - .75** | **.00** |
| 4) SAFE PLACES FOR BICYCLE AT SCHOOL |  |  |  |
| a) not important (ref.) | 1 | - | - |
| b) important | **.84*** | **.77 - .91** | **.00** |
| c) very important | **.89*** | **.82 - .96** | **.00** |
| 5) SAFE PLACES TO CROSS ROAD |  |  |  |
| a) not important (ref.) | 1 | - | - |
| b) important | 1.07 | .99 - 1.16 | .10 |
| c) very important | **1.14*** | **1.05 - 1.24** | **.00** |
| 6) PEOPLE TO WALK WITH |  |  |  |
| a) not important (ref.) | 1 | - | - |
| b) important | **.74*** | **.69 - .79** | **.00** |
| c) very important | **.64*** | **.60 - .70** | **.00** |
| 7) NOT WORRIED ABOUT BEING BULLIED / ATTACKED |  |  |  |
| a) not important (ref.) | 1 | - | - |
| b) important | **1.09*** | **1.01 - 1.18** | **.03** |
| c) very important | **1,14*** | **1.06 - 1.23** | **.00** |
| 8) SCHOOL LOCKERS |  |  |  |
| a) not important (ref.) | 1 | - | - |
| b) important | **1.16*** | **1.07 - 1.26** | **.00** |
| c) very important | **1.31*** | **1.21 - 1.41** | **.00** |
| 9) LIVING CLOSER TO SCHOOL |  |  |  |
| a) not important (ref.) | 1 | - | - |
| b) important | **.72*** | **.67 - .78** | **.00** |
| c) very important | **.39*** | **.36 - .42** | **.00** |
| 10) BETTER STREET LIGHTS |  |  |  |
| a) not important (ref.) | 1 | - | - |
| b) important | 1.00 | .93 - 1.07 | .98 |
| c) very important | .99 | .91 - 1.07 | .71 |
| sex^1^ | .92* | .87 - .97 | .00 |
| age | .95* | .93 - .97 | .00 |
| PA last 7 days^2^ | 1.05* | 1.03 - 1.06 | .00 |
| meeting WHO PA recommendation | 1.20* | 1.11 - 1.29 | .00 |
| FAS 1^3^ | .69* | .64 - .74 | .00 |
| FAS 2^4^ | .47* | .44 - .51 | .00 |
| Distance to school | .89* | .88 - .90 | .00 |

OR= odds ratio, CI= confidence interval, p= p-value, ref.= reference category, PA= physical activity, WHO= world health organization, FAS= family affluence scale

*significant results on α=95%are bolded

^1^reference category boys

^2^continuous (1-7 days of PA/week)

^3^reference category medium vs. low FAS

^4^reference category high vs. low FAS

**
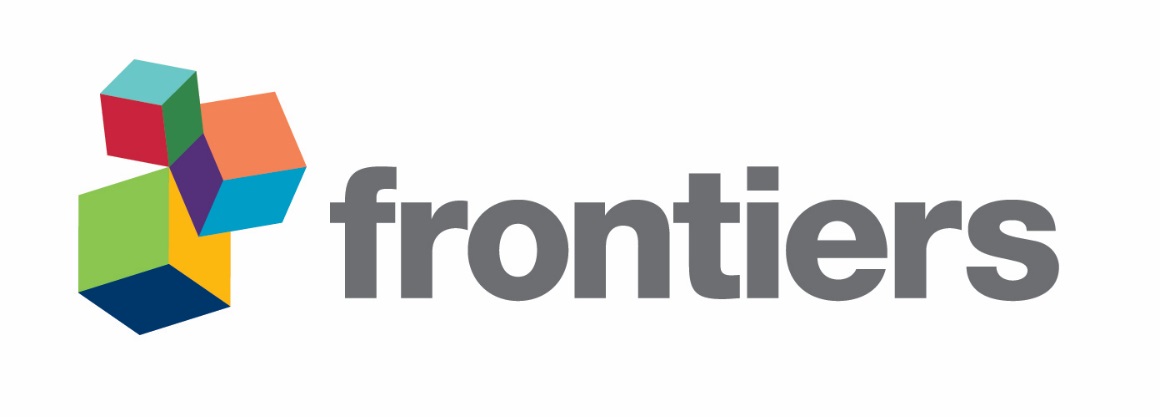
**
